# Complete genome sequence of Halorubrum pleomorphic virus 8

**DOI:** 10.12688/f1000research.179903.1

**Published:** 2026-04-30

**Authors:** Tatiana Demina, Elina Laanto, Hanna M. Oksanen

**Affiliations:** 1 Department of Microbiology, Faculty of Agriculture and Forestry, University of Helsinki, Helsinki, Uusimaa, Finland; 2 Department of Biological and Environmental Science and Nanoscience Center, University of Jyväskylä, Jyväskylä, Central Finland, Finland; 3 Department of Molecular and Integrative Biosciences, Faculty of Biological and Environmental Sciences, University of Helsinki, Helsinki, Uusimaa, Finland

**Keywords:** archaea, pleomorphic virus, viral genome, halophilic, solar saltern, Alphapleolipoviridae

## Abstract

Pleomorphic archaeal viruses are widespread and abundant in hypersaline environments and beyond. Still, a relatively low number of isolates are available for this viral group. Here, we present the complete genome sequence of Halorubrum pleomorphic viruses 8 (HRPV-8), which was previously isolated from the Samut Sakhon solar saltern, Thailand, on a *Halorubrum* species. The HRPV-8 genome was assembled into a circular 10,824-bp-long contig with a GC content of 63.2%. The genome annotation resulted in 12 open reading frames, including a VP4-like spike protein-encoding gene, which is a hallmark of pleomorphic archaeal viruses. Seven and three putative proteins in HRPV-8 were 99–100% identical to their counterparts in Halorubrum pleomorphic viruses 2 and 6 (HRPV-2 and HRPV-6), respectively, members of the genus *Alphapleolipovirus.* The average nucleotide identity values within these three viral genomes were 92–96%. According to VIRIDIC analyses of intergenomic similarities, HRPV-8 represents its own species. Thus, based on the observed pleolipoviral genome architecture and sequence similarities to other alphapleolipoviruses, we propose to classify HRPV-8 as a new species within the *Alphapleolipovirus* genus of the *Pleolipoviridae* family.

## Introduction

Archaeal viruses represent one of the least studied parts of the virosphere. Furthermore, the vast morphological and genetic diversity observed in the known archaeal viruses ( Krupovic et al. 2018) suggests that novel viruses await discovery. Hypersaline environments around the world have served as rich sources for isolating archaeal viruses ( Atanasova et al. 2012, 2015; Mizuno et al. 2019). So far, the majority of isolated halophilic archaeal viruses display tailed virus particles, while other virus morphotypes, such as pleomorphic, are currently represented by less isolates ( Atanasova et al. 2016; Demina and Oksanen 2020). Although originally found in hypersaline environments, pleomorphic archaeal viruses seem to be more widespread than first thought ( Medvedeva et al. 2023; Baquero et al. 2024). Currently (as of April 2026), three families within the realm *Pleomoviria* comprise pleomorphic archaeal viruses: *Pleolipoviridae* ( Liu et al. 2022) *, Thalassapleoviridae* ( Baquero et al. 2024; Varsani et al. 2025), and *Nanopleoviridae* ( Zhou et al. 2025), which include viruses associated with archaea within the classes Halobacteria and Archaeoglobi as well as the phylum Nanohaloarchaeota, respectively.

The family *Pleolipoviridae* includes three genera: *Alpha-*, *Beta-*, and *Gammapleolipoviridae* ( Liu et al. 2022). In addition, the *Delta-* and *Epsilon-* genera were proposed ( Alarcón-Schumacher et al. 2023). The currently ratified members of the family *Pleolipoviridae* demonstrate narrow host ranges within the class Halobacteria ( Liu et al. 2022), where they establish persistent, non-lytic infections and are thought to exit host cells via budding ( Pietilä et al. 2012; Svirskaitė et al. 2016; Alarcón-Schumacher et al. 2022), while some pleolipoviruses are temperate ( Liu et al. 2015; Wang et al. 2018; Atanasova et al. 2018; Dyall-Smith et al. 2021; Alarcón-Schumacher et al. 2023). Proviral regions and plasmids related to pleolipoviruses are widely found in archaea, suggesting their long-standing evolutionary relationships as well as the global abundance of these viruses and related mobile genetic elements ( Sencilo et al. 2012; Liu et al. 2015; Atanasova et al. 2018; Alarcón-Schumacher et al. 2023).

Pleolipoviral particles are pleomorphic membrane vesicles (typically 40–70 nm in diameter) which consist of two major proteins: a spike protein and an internal membrane protein, each of which can occur in one or two types ( Pietilä et al. 2012; El Omari et al. 2019). Interestingly, the relatively short genomes (7–17 nt or bp) vary in structure across the known isolates, and may be circular single-stranded (ss) DNA, circular double-stranded (ds) DNA (also with ss interruptions), or linear ds DNA molecules ( Pietilä et al. 2009; Roine et al. 2010; Sencilo et al. 2012). Overall nucleotide similarity between the genomes of pleolipoviruses is relatively low, but a cluster of typically five open reading frames (ORFs) are more conserved, including the gene encoding the spike protein (the VP4-like fusogen), which serves as the hallmark gene of this viral group ( Pietilä et al. 2012; Sencilo et al. 2012; El Omari et al. 2019; Alarcón-Schumacher et al. 2023).

Halorubrum pleomorphic virus 8 (HRPV-8) was isolated from an environmental sample collected at a solar saltern in Samut Sakhon, Thailand ( Atanasova et al. 2015). HRPV-8 produced turbid plaques on two *Halorubrum* strains originating from the same location. Although HRPV-8 was not sensitive to chloroform, transmission electron microscopy and the protein profile of the purified virions indicated that it is a pleomorphic virus ( Atanasova et al. 2015). Here, we present the complete genome sequence of HRPV-8 and suggest its classification within the genus *Alphapleolipovirus* of the family *Pleolipoviridae.*

## Methods

### Virus purification, nucleic acid extraction, and genome sequencing

HRPV-8 was grown aerobically on *Halorubrum* sp. SP3–3 in the modified growth medium (MGM) at 37 °C using the double-layer technique as described previously ( Atanasova et al. 2012, 2015). Confluent plates were used for preparing the virus stock which was used for virus purification. Viruses were purified using polyethylene glycol 6000 (Ubichem Cat No. KG P001-HS) and NaCl (Fisher Chemicals Cat No. S/3120/63) precipitation, followed by rate-zonal ultracentrifugation in sucrose (VWR Cat No. 27480.294) as described ( Atanasova et al. 2012). Nucleic acids were extracted from purified virus particles with PureLink™ Viral RNA/DNA Mini Kit (Thermo Fisher Scientific Cat No. 12280050). Genomic DNA was sequenced by Novogene (UK) using paired-end sequencing (2x150 bp) performed on the Illumina NovaSeq X platform, yielding a total of approximately 1 Gb of raw data.

### Genome assembly and analyses

Raw reads were trimmed using fastp v. 0.24.0 (−q 30 -l 50) ( Chen et al. 2018) and quality-checked with FastQC v. 0.12.1 ( https://www.bioinformatics.babraham.ac.uk/projects/fastqc/). Contigs were assembled using Spades v. 4.2.0 in the --meta mode ( Nurk et al. 2017), since the sample contained both viral and host DNA. BBTools Stats was used for assessing the assembly statistics ( sourceforge.net/projects/bbmap/). Among assembled contigs, the viral contig that was assigned to *Pleolipoviridae* with geNomad v. 1.7.6 (database v. 1.7) ( Camargo, Roux, et al. 2023b) was used for further analyses as the genome of HRPV-8. The whole genome sequence of HRPV-8 was searched against the NCBI core_nt database ( Sayers et al. 2024) and the IMG/VR v. 4 database ( Camargo, Nayfach, et al. 2023a) using BLASTn (E-value threshold of 1e-5) ( Altschul et al. 1990). Putative functions of the ORFs products in the HRPV-8 genome were predicted using Phold v. 0.2.0 ( Bouras et al. 2026) and PHROGs database ( Terzian et al. 2021), BLASTp searches against the NCBI nr database ( Altschul et al. 1990; Sayers et al. 2024), HHPred searches against the PDB_mmCIF70_25_May and SCOPe70_2.08 databases (probability threshold 95%) ( Söding et al. 2005; Gabler et al. 2020), as well as NCBI Conserved Domain searches against the CDD and COG databases (E-value threshold 1e-3) ( Marchler-Bauer et al. 2015). FastANI v. 1.33 ( Jain et al. 2018) was used to calculate average nucleotide identity (ANI) between viral genomes. Pairwise amino acid identities in HRPV-8, Halorubrum pleomorphic virus 2 (HRPV-2), and Halorubrum pleomorphic virus 6 (HRPV-6) proteins were calculated using EMBOSS Needle ( Madeira et al. 2024). Intergenomic similarities were calculated with the VIRIDIC web tool ( Moraru et al. 2020). The genome comparisons between HRPV-8 and other pleolipoviral genomes were visualized using Clinker v. 0.0.31 ( Gilchrist and Chooi 2021).

## Results

Raw data included 6,754,300 read pairs, which were trimmed to 6,183,469 read pairs. From trimmed reads, 461 contigs were assembled (min = 56 bp, max = 486,959 bp, N50 = 123,392 bp). From these contigs, only two were identified as viral by geNomad: one unclassified provirus (37,872 bp) and one virus assigned to the family *Pleolipoviridae* (10,879 bp). The latter one displayed terminal 55-bp-long repeats, which reflected the assembly artifact from a circular template, since the used k-mer was 55. The artificial nature of the repeat was confirmed by rerunning SPAdes with the k-mer size of 77 which produced the 10,901-bp-long pleolipoviral contig with different terminal repeats of 77 bp. Thus, one of the repeats was removed, resulting in a 10,824-bp-long circular sequence, which represents the complete genome sequence of HRPV-8. Its GC content is 63.2%.

Whole-genome searches against the NCBI core_nt database retrieved the best two hits to the Halorubrum pleomorphic viruses 2 and 6 (HRPV-2: 97% coverage, 95% nt identity; HRPV-6: 78% coverage, 96% nt identity) as well as multiple hits to the sequences of halophilic archaea and other pleolipoviruses, albeit with significantly lower coverage (≤37%) ( Table 1). The ANI values between HRPV-8 versus HRPV-2 and HRPV-8 versus HRPV-6 were 95.6% and 91.5%, respectively. For comparison, the ANI between HRPV-2 and HRPV-6 is 94.7%. Searching against the IMG/VR v. 4 database with the HRPV-8 whole genome as a query retrieved hits only to HRPV-2 and HRPV-6. The VIRIDIC analysis of intergenomic similarities between HRPV-8, HRPV-2, HRPV-6 and other current members of the *Alphapleolipovirus* genus suggested that HRPV-8 is a separate species ( Figure 1).

**Table 1.** Best BLASTn hits of the HRPV-8 genome to viral genomes.

Hit *	Coverage, %	Nt identity, %	GenBank acc no	Reference
Halorubrum pleomorphic virus 2 (HRPV-2)	97	95	NC_017087	( Sencilo et al. 2012)
Halorubrum pleomorphic virus 6 (HRPV-6)	78	96	NC_017089	( Sencilo et al. 2012)
Halorubrum virus Humcor2	35	74	MW344764	( Dyall-Smith et al. 2021)
Halorubrum pleomorphic virus 9 (HRPV9)	11	67	NC_048160	( Atanasova et al. 2018)
Tebenquiche pleomorphic virus 1 (Tebi-PV1)	7	72	PX315772	( Queiss et al. 2025)
Halorubrum pleomorphic virus 3 (HRPV-3)	6	70	NC_017088	( Sencilo et al. 2012)
Halorubrum pleomorphic virus 1 (HRPV-1)	6	69	NC_012558	( Pietilä et al. 2009)
Haloferax virus Halfgib1	6	67	MW344766	( Dyall-Smith et al. 2021)
Halorubrum pleomorphic virus 13 (HRPV13)	5	68	OM747534	( 宋珊珊 et al. 2022)

* the NCBI core_nt database; E-value threshold of 1e-5; search dated 29.1.26.

**Figure 1.**
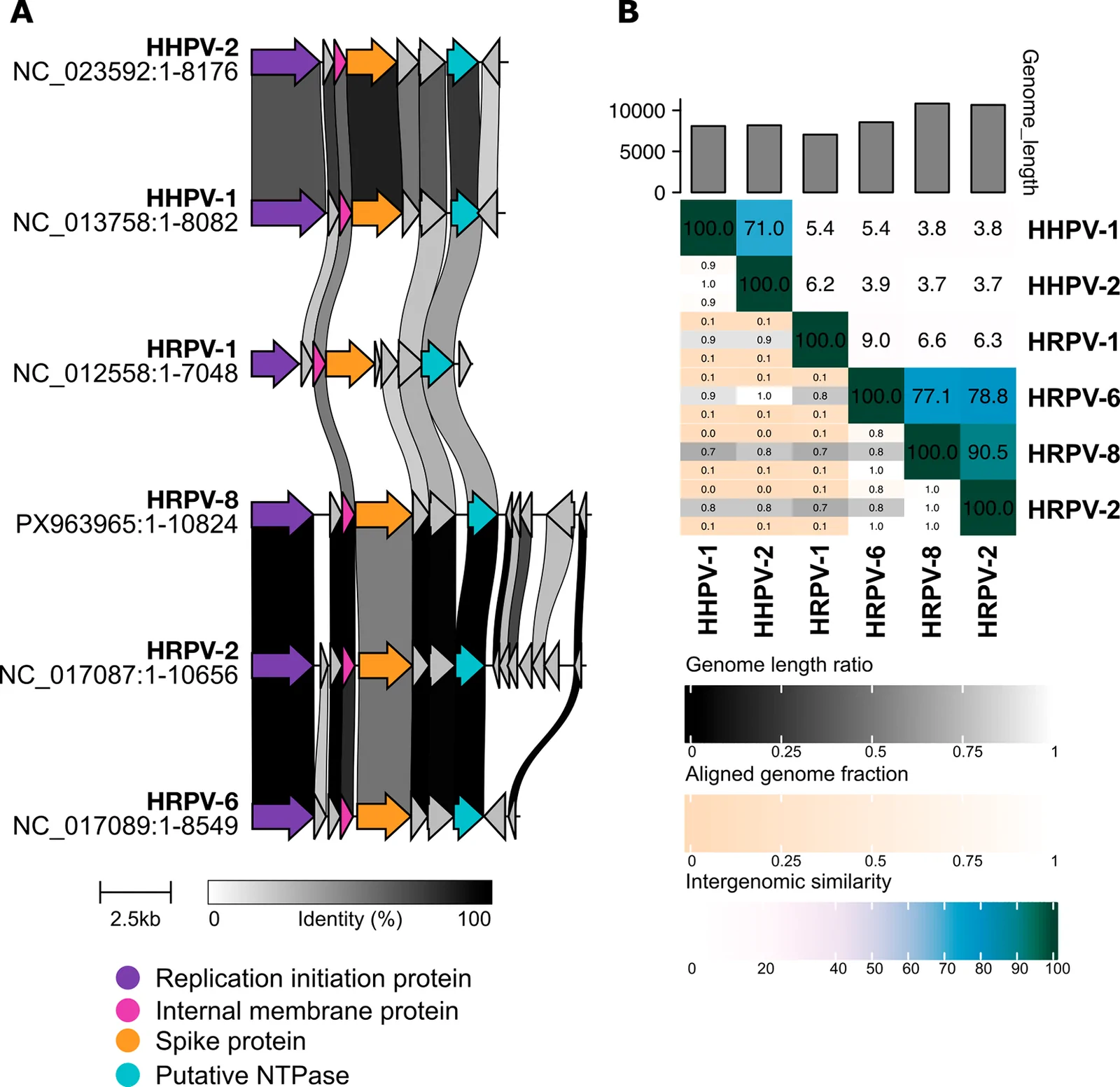
The genome of HRPV-8 and pleolipoviruses that are currently classified within the genus *Alphapleolipovirus* of the family *Pleolipoviridae.* (A) Genome comparisons were visualized with Clinker. ORFs are colored based on annotations (see the color code, ORFs with unknown functions are shown in grey). Shading of black between ORFs shows % protein identity (see the inset) between ORF products. (B) Intergenomic similarities visualized as a heatmap with VIRIDIC.

HRPV-8 genome annotation with Phold resulted in 12 predicted ORFs, most of which encoded gene products of unknown functions ( Table 2). Consistent with the observed ANI values, seven proteins were (almost) identical (99–100% amino acid identity) in HRPV-8 and HRPV-2 ( Table 2, Figure 1). Three of these were also 99–100% identical to their counterparts in HRVP-6. Based on the Phold prediction, Conserved Domain search, as well as similarities to other pleolipoviruses, gp1 was annotated as a putative DNA replication initiation protein in HRPV-8. gp2 was predicted as a single-stranded DNA binding protein by Phold. gp3 was predicted to be an internal membrane protein, as it was similar to such proteins in other pleolipoviruses. HHPred searches and observed similarities to counterparts in HRPV-2 and HRPV-6 suggested that gp4 was a spike protein (VP4-like). gp5 was annotated as an ATPase based on the Phold prediction, HHPred and Conserved Domain searches, as well as similarities to other pleolipoviruses. gp12 had multiple hits to antitoxin and DNA-binding proteins in HHPred searches, while Conserved Domain search against the COG database recruited a hit to DNA gyrase/topoisomerase IV, subunit A (Replication, recombination and repair, COG0188).

**Table 2.** Genome annotation of HRPV-8.

ORF *	Gene product	Start, nt	End, nt	Strand	Phold hit to PHROG category, no	Putative function	Amino acid identity (%) to a counterpart in	
							HRPV-2	HRPV-6
ORF1	gp1	1	1,968	forward	713	DNA replication initiation	99.5	98.9
ORF2	gp2	2,494	2,877	forward	4932	ssDNA binding protein	100	100
ORF3	gp3	2,877	3,278	forward	5900	Internal membrane protein	100	85.7
ORF4	gp4	3,302	5,119	forward	2231	Spike protein (membrane fusion)	59.1	54.9
ORF5	gp5	5,128	5,658	forward	1938	Unknown	99.4	99.4
ORF6	gp6	5,655	6,491	forward	No hit	Unknown	96.4	93.5
ORF7	gp7	6,885	7,838	forward	1675	ATPase	99.4	97.5
ORF8	gp8	8,089	8,286	reverse	No hit	Unknown	98.5	NA **
ORF9	gp9	8,283	8,549	reverse	No hit	Unknown	36.8	NA
ORF10	gp10	8,542	8,910	reverse	No hit	Unknown	77.9	NA
ORF11	gp11	9,384	10,265	reverse	No hit	Unknown	34.9	NA
ORF12	gp12	10,422	10,646	reverse	24852	DNA gyrase/topoisomerase subunit	100	95.9

* ORFs were numbered starting from the replication initiation protein-encoding ORF, consistently with other alphapleolipoviruses

** NA, not applicable (in case of no counterparts)

## Conclusions

HRPV-8 genome sequence is highly similar to those of the viruses HRPV-2 and HRPV-6, which are currently classified within the *Alphapleolipovirus* genus of the *Pleolipoviridae* family as the species of *Alphapleolipovirus thailandense* and *Alphapleolipovirus samutsakhonense*, respectively. While a few proteins were (almost) identical in HRPV-8, HRPV-2, and HRPV-6, the VP4-like spike proteins (a hallmark of pleomorphic archaeal viruses) share only 55–63% amino acid identities in these three viruses. The VIRIDIC-based comparisons of alphapleolipoviruses suggest that HRPV-8 is a separate species. Based on the observed similarities, we propose to classify HRPV-8 as a new member of the *Alphapleolipovirus* genus. The HRPV-8 sequence presented here contributes to resolving the genetic diversity of pleomorphic archaeal viruses, highlighting the mosaicisms of their genomes.

## Data Availability

NCBI GenBank: HRPV-8 complete genome sequence [HPRV-8]. Accession number PX963965.
