# Supplementary material for: Complete Genome Sequence of Halorubrum Pleomorphic Virus 8
Source: F1000Res. 2026 Oct 15;15:654. Originally published 2026 Apr 30. [Version 3] doi: 10.12688/f1000research.179903.3 (PMC13631441; doi:10.12688/f1000research.179903.3)
Supplement: Peer review history

# Reviewer report: Reviewer response for version 2

**DOI:** 10.5256/f1000research.209050.r516734

**Published:** 2026-10-01

**Authors:** Ingrida Olendraitė

**Affiliations:** 1 EMBL-EBI, Cambridgeshire, UK

The article describes a new complete genome sequence of a virus found in halophylic Halorubrum archaea. Researchers have sequenced the sample, did quality control and assembly of the genome and compared with the existing sequences in the databases and propose the new virus species within the Alphapleolipovirus genus of Pleolipoviridae family. 

 Below are the comments, not all need to be addressed. Work has been done well and suggestions are merely for clarity and potentially improvements rather than things that need to be fixed for the indexing. 

 low number of isolates

- Isolates can be understood as members of the same species - but you have a unique species; so maybe a low number of isolated or described species? 
- But this term might be more widely accepted in archaeal community, which I have no knowledge of. 

Halorubrum pleomorphic viruses 8 (HRPV-8)

- Not viruses - should be “virus”, correct?

Seven and three putative proteins in HRPV-8 were 99–100% identical to their counterparts in Halorubrum pleomorphic viruses 2 and 6 (HRPV-2 and HRPV-6), respectively,

- seven and three >...< respectively - a very big gap between the HRPV-2 and -6 and the first words; reads a bit clunky. 

- Additionally - 12 ORFs doesn’t mean 12 proteins (peptides); so 7 and 3 here do not really mean much (unless you are talking about ORFs), because we don’t know how many are not identical. In short: ORF is not the same as protein. 

The average nucleotide identity values within these three viral genomes were 92–96%.

- Is this for a full genome? 

Overall nucleotide similarity between the genomes of pleolipoviruses is relatively low,

- It would be interesting to see these numbers (what is relatively low?) - as for your new virus you report protein and nucleotide identity which is quite high  (99-100 or 92-96) - this could be very useful in the introduction to provide context.

Halorubrum pleomorphic virus 8 (HRPV-8) was isolated from an environmental sample collected at a solar saltern in Samut Sakhon, Thailand (Atanasova et al., 2015). HRPV-8 produced turbid plaques on two Halorubrum strains originating from the same location. Although HRPV-8 was not sensitive to chloroform, transmission electron microscopy and the protein profile of the purified virions indicated that it is a pleomorphic virus (Atanasova et al., 2015). Here, we present the complete genome sequence of HRPV-8 and suggest its classification within the genus Alphapleolipovirus of the family Pleolipoviridae.

- After finishing reading the introduction it is not clear if in 2015 the HRPV-8 had genome sequence or it was just isolated and some plaque assays etc done. This is important to assess the novelty of your work (and to understand if it's a new isolate of the same species or new species, or finally a complete genome etc). Whatever was lacking - it would be good to explicitly mention in the introduction. 

Methods

 value threshold of 1e-5

- It’s usually 10e-5

Pairwise amino acid identities in HRPV-8, Halorubrum pleomorphic virus 2 (HRPV-2), and Halorubrum pleomorphic virus 6 (HRPV-6) proteins were calculated using EMBOSS Needle (Madeira et al., 2024). I

- It would be useful to provide accessions here (not necessarily just in the results table) if you have for HRPV-2 and -6 - so it’s exactly clear what was compared. 

Results

 Raw data included 6,754,300 read pairs, which were trimmed to 6,183,469 read pairs.

- Filtered; trimming is from the start/end of a read - so base pairs would be trimmed and then some of the reads wouldn’t pass the length threshold or be discarded due to quality. So trimming is different action. 
- To improve readability you could just say  “After trimming and quality control, 6,183,469 reads remained and were assembled into 461 contigs (). 

terminal 55-bp-long repeats, which reflected the assembly artifact from a circular template,

- Repeats might arise due to assemblers and k-mer sizes for non-circular templates as well. I’d add some softening confidence word: which reflected the possible assembly artifact due to a circular template
- Do you know that this virus has a circular template for sure? Has it been shown before?  If yes - citation could be helpful here. 
- The 77-kmer is a strong support. I would love to see if there are any “split reads”  to show a read which would cover both contig ends. 
- Also by mapping back the reads you could see if coverage drops around the joined sequence or it’s similar coverage throughout. 

as well as multiple hits to the sequences of halophilic archaea

- So your sequence potentially has integrated into archaeal genomes? Does this sentence mean it’s matching archaeal genomes or viruses of halophilic archaea? From your table 1 it seems as only to archaeal viruses.

HRPV-8 genome annotation with Phold resulted in 12 predicted ORFs, most of which encoded gene products of unknown functions ( Table 2).

- 6 had unknown functions - so it’s half, not most 
- In the table, lengths of the ORFs in amino acids could be useful (easier to compute in mind than nucleotide coordinates) to understand if it’s polyproteins or peptides, especially when evaluating the coverage / identity. It can of course be inferred from nucleotide coordinates. 

As a next step ideas: 

1. structural comparisons of proteins with unknown functions?
2. It would be great to see genus + members of the family phylogenetic tree? Maybe of the hallmark gene if it’s too complex computationally otherwise? 

Species significance could have been described in a bit more detail.

Are the datasets clearly presented in a usable and accessible format, and the assembly and annotation available in an appropriate subject-specific repository?

Yes

Are sufficient details of the sequencing and extraction, software used, and materials provided to allow replication by others?

Yes

Are the rationale for sequencing the genome and the species significance clearly described?

Yes

Are the protocols appropriate and is the work technically sound?

Yes

Reviewer Expertise:

Novel virus discovery, metatranscriptomics, metagenomics, molecular virology, phylogenetics, taxonomy

I confirm that I have read this submission and believe that I have an appropriate level of expertise to confirm that it is of an acceptable scientific standard.

# Response

**Published:** 2026-10-08

**Authors:** Tatiana Demina

**Affiliations:** University of Helsinki, Helsinki, Uusimaa, Finland

We thank the reviewer for valuable comments and suggestions on how the work could be improved. We have revised the article based on these points, and below are our answers (A) to reviewer’s questions/comments (Q).

 Q: *low number of isolates*

*Isolates can be understood as members of the same species - but you have a unique species; so maybe a low number of isolated or described species?*

 A: We have changed wording to “a low number of isolated and described species”, as it reflects the original point indeed more accurately.

 ---

 Q: *Halorubrum pleomorphic viruses 8 (HRPV-8)*

*Not viruses - should be “virus”, correct?*

 A: Yes, the typo has been corrected.

 ---

 Q: *Seven and three putative proteins in HRPV-8 were 99–100% identical to their counterparts in Halorubrum pleomorphic viruses 2 and 6 (HRPV-2 and HRPV-6), respectively,*

*seven and three >...< respectively - a very big gap between the HRPV-2 and -6 and the first words; reads a bit clunky*.

 A: We have reduced the gap by moving “in HRPV-8” to the beginning of the sentence.

 ---

 Q: *Additionally - 12 ORFs doesn’t mean 12 proteins (peptides); so 7 and 3 here do not really mean much (unless you are talking about ORFs), because we don’t know how many are not identical. In short: ORF is not the same as protein.*

 A: In this case, 12 ORFs encoded 12 proteins, and to avoid confusion with the terms, we have revised the text: “The genome was predicted to encode 12 proteins, including ...”.

 ---

 Q: *The average nucleotide identity values within these three viral genomes were 92–96%.*

*Is this for a full genome?*

 A: These average nucleotide identity (ANI) values were obtained using FastANI, which calculates whole-genome ANI, “defined as mean nucleotide identity of orthologous gene pairs shared between two microbial genomes” (https://github.com/ParBliSS/FastANI).

 ---

 Q: *Overall nucleotide similarity between the genomes of pleolipoviruses is relatively low,*

*It would be interesting to see these numbers (what is relatively low?) - as for your new virus you report protein and nucleotide identity which is quite high  (99-100 or 92-96) - this could be very useful in the introduction to provide context.*

 A: We agree that additional context would be useful here. However, to support the statement quantitatively, nucleotide identity/similarity values would need to be calculated consistently across all pleomorphic viruses. Rather than introducing additional comparative data, we removed the phrase about the low similarity, as these additional analyses would be beyond the scope of the Introduction.

 ---

*Q: <…> Here, we present the complete genome sequence of HRPV-8 and suggest its classification within the genus Alphapleolipovirus of the family Pleolipoviridae.*

*After finishing reading the introduction it is not clear if in 2015 the HRPV-8 had genome sequence or it was just isolated and some plaque assays etc done. This is important to assess the novelty of your work (and to understand if it's a new isolate of the same species or new species, or finally a complete genome etc). Whatever was lacking - it would be good to explicitly mention in the introduction.*

 A: The isolate was obtained and first reported in the study by Atanasova et al in 2015, but its genome sequence stayed unknown back then. In this study, we sequenced and annotated the genome of the that same isolate reported earlier. The isolate represents its own species (for which no other isolates are available), and its classification is currently under processing by ICTV. We have clarified the text by stating that “In this study, we sequenced and annotated the genome of this HRPV-8 isolate”.

 ---

 Q: *value threshold of 1e-5*

*It’s usually 10e-5*

 A: We have used the default BLASTn value at the IMG/VR v.4 ( https://img.jgi.doe.gov/cgi-bin/vr/main.cgi?section=WorkspaceBlast&page=viralform), which is 1e-5.

 ---

 Q: *Pairwise amino acid identities in HRPV-8, Halorubrum pleomorphic virus 2 (HRPV-2), and Halorubrum pleomorphic virus 6 (HRPV-6) proteins were calculated using EMBOSS Needle (Madeira et al., 2024). I*

*It would be useful to provide accessions here (not necessarily just in the results table) if you have for HRPV-2 and -6 - so it’s exactly clear what was compared.*

 A: Indeed, accession numbers add clarity, and they have been added to Table 2.

 ---

 Q: *Raw data included 6,754,300 read pairs, which were trimmed to 6,183,469 read pairs.*

*Filtered; trimming is from the start/end of a read - so base pairs would be trimmed and then some of the reads wouldn’t pass the length threshold or be discarded due to quality. So trimming is different action.*

*To improve readability you could just say  “After trimming and quality control, 6,183,469 reads remained and were assembled into 461 contigs ().*

 A: We have changed the wording according to this suggestion.

 ---

 Q: *terminal 55-bp-long repeats, which reflected the assembly artifact from a circular template,*

*Repeats might arise due to assemblers and k-mer sizes for non-circular templates as well. I’d add some softening confidence word: which reflected the possible assembly artifact due to a circular template*

*Do you know that this virus has a circular template for sure? Has it been shown before?  If yes - citation could be helpful here.*

*The 77-kmer is a strong support. I would love to see if there are any “split reads”  to show a read which would cover both contig ends.*

*Also by mapping back the reads you could see if coverage drops around the joined sequence or it’s similar coverage throughout.*

 A: We have no experimental evidence of the circular nature of the HRPV-8 genome, so we have added some softening words to these lines, as suggested. We have also additionally mapped reads to the HRPV-8 sequence linearized at different positions and observed read coverage across the entire genome (no uncovered positions) and no apparent coverage drop around the original contig ends. These results have been added.

 ---

 Q: *as well as multiple hits to the sequences of halophilic archaea*

*So your sequence potentially has integrated into archaeal genomes? Does this sentence mean it’s matching archaeal genomes or viruses of halophilic archaea? From your table 1 it seems as only to archaeal viruses.*

 A: Pleolipoviral sequences are often found as putative proviruses in archaeal genomes. We have moved mentioning the hits to archaeal genomes into a separate sentence to distinguish them from the matches to viral genomes. Table 1 contains hits only to viral genomes (as stated in its heading), since the focus of this study is on pleomorphic virus isolates rather than proviral elements in archaeal genomes.

 ---

 Q: *HRPV-8 genome annotation with Phold resulted in 12 predicted ORFs, most of which encoded gene products of unknown functions ( Table 2).*

*6 had unknown functions - so it’s half, not most*

 A: Thanks for noticing this, it has been corrected.

 ---

 Q: *In the table, lengths of the ORFs in amino acids could be useful (easier to compute in mind than nucleotide coordinates) to understand if it’s polyproteins or peptides, especially when evaluating the coverage / identity. It can of course be inferred from nucleotide coordinates.*

 A: The putative protein lengths in amino acids were added to the table.

 ---

*As a next step ideas:*

 Q: *structural comparisons of proteins with unknown functions?*

 A: In this work, we used sequence-and structure-based predictions as well as comparison to other pleolipoviruses for predicting putative functions HRPV-8 proteins, but indeed, further comparative structural analyses and experimental validation will be needed to determine the functions of the remaining uncharacterized proteins. We have added this point to the Conclusions.

 ---

 Q: *It would be great to see genus + members of the family phylogenetic tree? Maybe of the hallmark gene if it’s too complex computationally otherwise?*

 The phylogenetic tree of current and newly proposed species within the Pleolipoviridae family is already made as part of the taxonomic proposal that has been recently submitted to ICTV. It will be available from the ICTV website once processed.

 ---

 Q: *Species significance could have been described in a bit more detail.*

 A: The HRPV-8 genome expands the currently limited collection of cultivated pleomorphic archaeal viruses and provides an additional reference for studying species-level diversity within the family Pleolipoviridae as well as pleolipovirus evolution in general. We have added some text along these lines to the Conclusions.

# Reviewer report: Reviewer response for version 1

**DOI:** 10.5256/f1000research.198462.r481714

**Published:** 2026-06-01

**Authors:** Jimmy Saw

**Affiliations:** 1 George Washington University, Washington, USA

**Summary**

 The authors describe the recovery of a complete genomic sequence of poorly characterized archaeal virus belonging to the *Pleolipoviridae *family that infects halophilic archaea of genus *Halorubrum*. After comparison with other known viruses of the same family, they determined that the virus they assembled belong to a new species within the genus of *Alphapleolipovirus* based on average nucleotide identities.

**Review**

 The manuscript is clearly written, easy to read, and I have no major issues with the manuscript. Methods described in the manuscript appear to be sound and reproducible. However, there are a few minor issues to be fixed.

 First, I believe, it should be 7-17kbp here – “Interestingly, the relatively short genomes ( **7–17 nt or bp**)”.

 Second, raw metagenomic reads should be submitted to NCBI SRA or EBI ENA databases to allow reproducibility of assembly/binning workflow.

Are the datasets clearly presented in a usable and accessible format, and the assembly and annotation available in an appropriate subject-specific repository?

Yes

Are sufficient details of the sequencing and extraction, software used, and materials provided to allow replication by others?

Yes

Are the rationale for sequencing the genome and the species significance clearly described?

Yes

Are the protocols appropriate and is the work technically sound?

Yes

Reviewer Expertise:

Microbial ecology and genomics.

I confirm that I have read this submission and believe that I have an appropriate level of expertise to confirm that it is of an acceptable scientific standard.

# Response

**Published:** 2026-08-20

**Authors:** Tatiana Demina

**Affiliations:** University of Helsinki, Helsinki, Uusimaa, Finland

We thank the reviewer for the comments, which we have addressed in the revised manuscript: a typo in the pleolipoviral genome size range has been corrected, and raw reads from HRPV-8 whole genome sequencing have been deposited in the European Nucleotide Archive (ENA).
